# Heart rate and blood pressure variability, and baroreflex sensitivity, during heat exposure in persons with type 2 diabetes and hypertension

**DOI:** 10.1371/journal.pone.0361367

**Published:** 2026-10-27

**Authors:** Mojdeh Rafieian, Erlend Hoftun Farbu, Anje Christina Höper, Rasmus Valtonen, Mika Kallio, Henna Hyrkäs-Palmu, Craig Crandall, Tiina Maria Ikäheimo

**Affiliations:** 1 Department of Community Medicine, Faculty of Health Sciences, UiT the Arctic University of Norway, Tromsø, Norway; 2 Department of Occupational and Environmental Medicine, University Hospital of North Norway, Tromsø, Norway; 3 Research Unit of Biomedicine and Internal Medicine, University of Oulu, Oulu, Finland; 4 Medical Research Center Oulu, Oulu University Hospital and University of Oulu, Oulu, Finland; 5 Clinical Neurophysiology, Department of Diagnostics, Oulu University Hospital, Oulu, Finland; 6 Clinical Neurophysiology, Research Unit of Health Sciences and Technology, Faculty of Medicine, Medical Research Center, University of Oulu, Oulu, Finland; 7 Research Unit of Population Health, University of Oulu, Oulu, Finland; 8 Institute for Exercise and Environmental Medicine, Texas Health Presbyterian Hospital Dallas and University of Texas Southwestern Medical Center, Dallas, Texas, United States of America; Japanese Academy of Health and Practice, JAPAN

## Abstract

The autonomic nervous system regulates cardiovascular homeostasis during stressors such as heat, but the effects of type 2 diabetes (T2D) with hypertension on such responses are unclear. Twenty male participants (10 with type 2 diabetes and hypertension and 10 age-matched healthy controls; mean ages 64 ± 4.14 and 63 ± 5.42 years) underwent a 90-minute whole-body heat exposure at 40 °C and 50% relative humidity. Heart rate variability (HRV), baroreflex sensitivity (BRS), and blood pressure variability (BPV) were assessed before, during and after the heat exposure. Heat exposure increased heart rate in both groups but this response was less in T2D (5.43 bpm; ~ 7% increase) versus controls (13.96 bpm; ~ 22% increase; p = 0.014). Similarly, reductions in HRV indices to heat stress were generally smaller in magnitude among participants with T2D. For example, high-frequency power (HF RR) decreased by 31.21 ms² (~54.5% reduction) in T2D because of heat exposure, compared with a reduction of 179.35 ms² (~74% reduction) in controls (p = 0.038). Further, LF BRS decreased by 1.07 ms/mmHg (~25% reduction) vs. 3.96 ms/mmHg (~51% reduction) (p = 0.021), HF BRS by 1.58 ms/mmHg (~35% reduction) vs. 4.15 ms/mmHg (~62% reduction) (p = 0.034), and mean BRS by 1.18 ms/mmHg (~26% reduction) vs. 4.16 ms/mmHg (~58% reduction) (p = 0.010) in those with T2D versus controls, respectively. Low-frequency power (LF RR), LF/HF ratio and BPV parameters also showed smaller (though nonsignificant) changes in T2D. Overall, combined T2D and hypertension produced blunted autonomic responses to short-term heat exposure, potentially increasing vulnerability to temperature extremes.

## Introduction

The autonomic nervous system (ANS) plays a central role in maintaining cardiovascular homeostasis, particularly under conditions of physiological stress such as thermal exposure [1]. In individuals with type 2 diabetes (T2D), autonomic dysfunction is a well-documented complication, often preceding cardiovascular disease [2]. Dynamic measures of autonomic function, and related indices, such as heart rate variability (HRV), blood pressure variability (BPV), and baroreflex sensitivity (BRS) provide critical insights into the adaptability of cardiovascular regulation [3]. HRV represents temporal fluctuations between successive heartbeats and reflects autonomic modulation of cardiac function [4]. BPV represents spontaneous fluctuations in arterial pressure and provides insight into sympathetic vasomotor tone and vascular reactivity [5]. Baroreflexes are important mechanisms for short-term regulation of blood pressure, acting through vagal and sympathetic pathways and influencing both heart rate and vascular tone. These responses can be quantified by the assessment of BRS [6]. Indeed, HRV and BRS are valuable indicators for detecting early signs of autonomic imbalance, a condition frequently associated with metabolic syndrome (MetS) and T2D [7]. This imbalance, typically marked by increased sympathetic activity and reduced parasympathetic tone, is closely linked to insulin resistance. Such dysfunction not only impairs cardiovascular regulation but also elevates long-term cardiovascular risk [7,8].

Thermal stress—induced by environmental heat exposure —challenges cardiovascular stability through complex interactions between autonomic and non-autonomic mechanisms [9]. While the autonomic nervous system primarily adjusts heart rate, skin blood flow, and sweating to maintain thermal balance, other factors such as endothelial function and metabolic factors modulate these physiological responses [10–12]. In healthy individuals, this integrated system operates with a high degree of flexibility, enabling rapid adjustment and efficient recovery from stress [13,14]. Conversely, in individuals with T2D, impairments in both autonomic and vascular components can hinder these adaptive responses [15,16]. Autonomic function during thermal stress has mainly been investigated in healthy individuals and in populations with clinical conditions such as cardiovascular disease and hypertension [17–19]. While previous research on heat exposure in persons with T2D has primarily focused on HRV responses [20,21], to our knowledge no studies have addressed BRS and BPV, which are markers of cardiovascular regulation. Therefore, a greater understanding of the mechanisms modulating cardiovascular regulation in response to these environmental stressors in this vulnerable population is needed. In our previous study we observed blunted cardiovascular responses to whole-body heat exposure in individuals with T2D [22]. Building on these findings, the present study examined autonomic responses (HRV, BPV and BRS) in the same population at baseline, during environmental heat exposure, and during follow-up. We hypothesized that, compared to healthy controls, individuals with T2D would exhibit attenuated changes in indices of autonomic function during environmental heat exposure.

## Materials and methods

### Ethical approval

The laboratory study took place at the University of Oulu, Finland, during the winter and spring seasons of 2021. Approval for the study was obtained from the Ethics Committee of the Oulu University Hospital District (EETTMK:199/2016), and the trial was registered in the Clinical Trials database with the identifier NCT04698200. Furthermore, the Regional Committee for Medical Research Ethics in Norway approved the analysis of the data (Ref.nr. 2023/672049), and the Norwegian Agency for Shared Services in Education and Research reviewed the documentation related to personal data processing (Ref. No. 800577). The study adhered to the ethical principles of the Declaration of Helsinki, and all participants gave written informed consent prior to participation.

### Participants

The recruitment process for this study was explained in our previous paper [22]. In brief, 20 men aged 40–79 living in Northern Ostrobothnia were randomly selected from the Finnish Population Register in 2019. Overall recruitment was carried out between 1.11. 2019–30.6. 2020. Following eligibility screening and exclusions, 10 participants with combined T2D and hypertension and 10 matched healthy control subjects were included in the final study sample. Inclusion criteria for the T2D group included a disease duration of at least 2 years, diagnosed hypertension, HbA1c levels between 7–10% (53–86 mmol/mol), and no history of smoking or active retinopathy. Control participants were free from T2D, hypertension, and smoking history. Groups were matched as closely as possible for age and BMI. The final samples for the T2D and control groups were 8 and 10 participants, respectively, at baseline. Due to technical issues affecting signal quality during heat exposure, the number of participants included in heat-exposure analyses was reduced to 7 and 9, respectively.

### Assessment of baseline characteristics prior to the controlled experiments

Each participant completed a questionnaire regarding health, lifestyle factors, and medication use. Height and weight were recorded, and body fat percentage was estimated using a four-site skinfold measurement method (triceps, biceps, subscapular, and suprailiac) [23]. Peripheral artery disease status was evaluated using the ankle-brachial index (ABI), which involved measuring systolic pressures from both the arms and ankles. The ABI was calculated as the ratio of ankle systolic pressure to brachial systolic pressure, determined separately for each leg, with the lower of the two values used as the participant’s result.

Thermal sensitivity and pain detection were assessed using the Medoc Pathway CHEPS system, which is a widely used and validated quantitative sensory testing method [24]. Cold and heat detection thresholds were measured separately on the thenar eminence (hand) and the plantar surface (foot) by applying temperature stimuli via a thermode that gradually decreased or increased from a reference temperature of 30 °C, with limits of 0 °C for cold and 50 °C for heat. Pain detection was evaluated by increasing heat intensity until participants reported pain. Additionally, the monofilament test was conducted to screen for neuropathy and loss of protective sensation. A 10-gram filament was applied perpendicularly until it bent at three specific points on each foot to assess sensory response. Detection at all points indicated normal sensation, while failure to sense the filament at any point suggested neuropathic impairment. Skin temperature was presented over 10 minutes of baseline, at 60 minutes of heat exposure, and the first 10 minutes of follow-up.

#### The autonomic nervous system (ANS) parameters.

Baseline autonomic nervous system measurements were performed on a separate day relative to the heat stress trial at the University Hospital of Oulu before the experimental study. ANS function was assessed using the Valsalva maneuver, the 30:15 ratio during passive tilt, paced breathing, and 5-minute resting heart rate variability (HRV). The Valsalva test was performed seated, using a 40-mm Hg expiratory strain for 15 seconds, measured using a pressure gauge attached to a 5- to 10-mL plastic syringe and repeated until two consistent blood pressure responses were obtained. Heart rate and blood pressure were continuously monitored using an electrocardiography and a noninvasive beat-to-beat blood pressure device, respectively. The Valsalva ratio (VR) was calculated by dividing the maximum heart rate during the blood pressure drop by the minimum heart rate following the subsequent rebound [25]. Blood pressure recovery time (PRT) was defined as the interval from the nadir of systolic blood pressure at the end of Phase III to the return to baseline levels. This measure reflects the time required for sympathetic vasoconstriction to normalize blood pressure after transient hypotension [26,27]. The “30:15 ratio” was calculated by dividing the longest RR interval, occurring around beat 30 (within beats 20–40), by the shortest RR interval, occurring around beat 15 (within beats 10–20), following a rapid passive upright tilt, which reflects the vagal responses (2 seconds to 80°) [25].

During paced breathing, R–R intervals were analyzed by calculating the ratio of the longest (during expiration) to the shortest (during inspiration) R–R interval over five consecutive breathing cycles [25]. This test was performed twice, and the higher median R–R interval ratio from the two trials was recorded as the “maximum–minimum ratio.” HRV was analyzed using electrocardiographic recordings obtained during a 5-minute resting period in the supine position, following a 30-minute rest period preceding the tests. Analyzed indices of HRV included mean RR interval, SDNN, RMSSD, and frequency-domain indices (LF, HF, LF/HF ratio) reflecting sympathovagal balance. More detailed procedures and calculations are provided in S1 File.

### Controlled experiments

The complete methodology for this study has been explained in our previous paper [22]. In brief, all measurements started at the same time in the morning, with participants advised avoiding strenuous physical activity for 24 hours, alcohol for 48 hours, and caffeine for 2 hours prior to testing. Each was instructed to eat breakfast as usual and continue their medications. Hydration status was assessed from urine specific gravity before the experiments using a PAL-10S refractometer (ATAGO, Tokyo, Japan), confirming that the participants were not dehydrated (urine specific gravity <1.020). Blood glucose levels were measured before and after the experiments using an Accu-Chek Aviva glucose meter (Roche Diabetes Care, Inc), confirming no indications of hypoglycaemia. During heat trials, participants wore light clothing—shorts, socks, t-shirt, and sandals—with an insulation value of approximately 0.26 clo [28].

For the baseline assessment, participants remained seated for 30 minutes in a neutral (25°C) environment. After this period, they were exposed to whole-body heat (40°C; 50% relative humidity) via a climate chamber for 90 minutes. The participants were in the seated position throughout this exposure. Following the heat exposure, participants were again seated for 30 minutes in the neutral environment (25°C) for the follow-up assessment (Fig 1). The heat exposure was intended to induce sweating, while stimulating the cardiovascular system and provoking cardiovascular strain.

**Fig 1:**
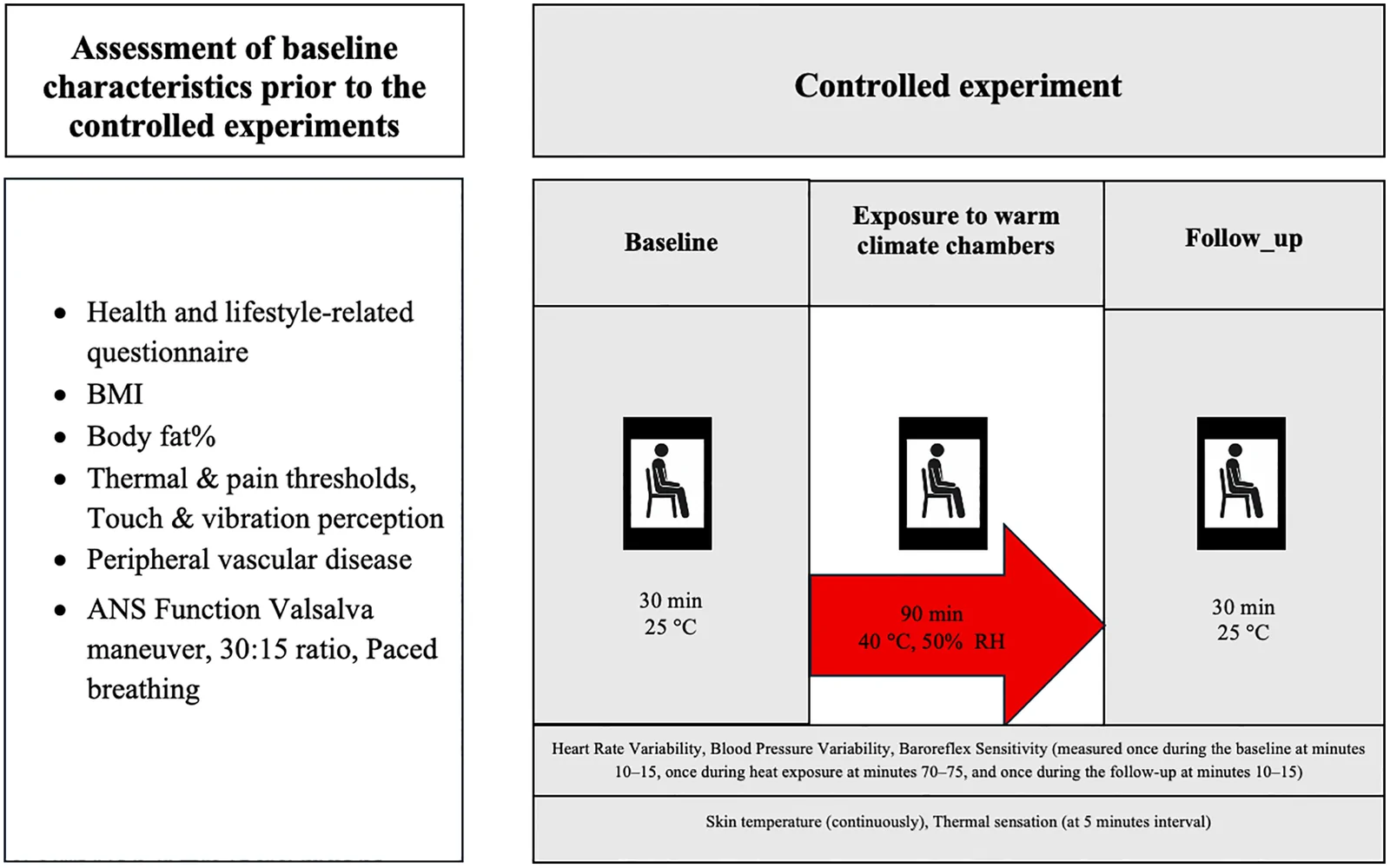
Diagram of study design. Baseline testing was followed by heat exposure, and then a post heat exposure follow-up period. Autonomic and cardiovascular responses were assessed at each time points. Abbreviations; BMI, body mass index, ANS, autonomic nervous system; RH, relative humidity; °C, degrees Celsius.

#### Skin temperature and thermal sensations.

Skin temperature was recorded using thermistors (NTC DC95, Digi-Key, Thief River Falls, MN, USA) attached at eight different sites, with readings registered at 1-min intervals via temperature data loggers (SmartReaderPlus; Acr Systems Inc., BC, Canada). The mean skin temperature (Tsk) was calculated using the following weighted formula: Tsk = [0.07 forehead + 0.175 right scapula + 0.175 left upper chest + 0.07 right arm + 0.07 left arm + 0.05 left hand + 0.19 right anterior thigh + 0.2 left calf], according to ISO 9886 [29]. Participants reported their thermal sensations based on subjective judgment scales (ISO 10551) [30] at baseline, exposure and follow-up. Skin temperature data are presented during the final 10-minutes of pre-heating baseline, at 60 minutes into the heat exposure, and during the first 10 minutes of follow-up (Fig 1).

#### The autonomic nervous system (ANS) parameters.

Heart rate variability. Heart rate variability (HRV) was analyzed using electrocardiographic recordings. The average R–R interval (RRI) represented the mean duration between consecutive heartbeats. The standard deviation (SD) of R–R intervals provided a measure of overall variability in heart rate. The root mean square of successive differences (RMSSD) between adjacent R–R intervals was calculated as an indicator of short-term, high-frequency variations primarily influenced primarily by parasympathetic activity. From these recordings, both time-domain and frequency-domain parameters were calculated. In the frequency domain, low-frequency (LF) and high-frequency (HF) components were expressed in normalized units (nu) to allow for meaningful comparison between sympathetic and parasympathetic contributions. The LF component, typically associated with both sympathetic and parasympathetic modulation, and the HF component, predominantly reflecting parasympathetic tone, were used to derive the LF/HF ratio reflecting sympathovagal balance [4,31].

Blood pressure variability and baroreflex sensitivity. Continuous arterial blood pressure (BP) was recorded using a non-invasive device based on the volume clamp method (Nexfin, BMEYE Medical Systems, Netherlands). A cuff was fitted to the right index finger, with the arm supported at heart level. A height sensor, positioned at the level of the heart, allowed the system to automatically adjust for hydrostatic pressure. To maintain accuracy of continuous BP measurement, the automatic calibration (physiocal) was performed at least once every 70 seconds. Respiration was monitored with a piezoelectric belt (PneumoTrace, ADInstruments, Australia). Continuous BP, respiratory, and ECG signals were digitized at a 1 kHz sampling rate using an analog-to-digital converter (PowerLab/8SP, ADInstruments, Australia) and processed with LabChart software (version 7.3.2, ADInstruments, Australia). Data from continuous BP, respiration, and ECG recordings were analyzed using with a custom-made Matlab-based (MathWorks, Inc., Natick, MA, USA) software. Ectopic or irregular beats were manually excluded from the signals and substituted with the local average. Missing data segments caused by automatic calibration were corrected using linear interpolation. From the processed signals, time series of RR intervals and beat-to-beat systolic BP were derived. Total BP variability was estimated as the standard deviation of systolic BP, calculated over the full 5-minute measurement period.

For spectral analysis, very low-frequency components (<0.04 Hz) were removed using the Savitzky–Golay detrending method. Spectral estimates were then calculated for stable 5-minute segments taken from the end of both baseline, heat exposure recovery phases, focusing on the LF (0.04–0.15 Hz) and HF (0.15–0.4 Hz) ranges of HR and BP variability, using FFT with Welch’s method (sequence length 128, 50% overlap).The spectral estimate of spontaneous BRS in the LF band was obtained using the alpha method [32], defined as 𝐵𝑅𝑆 = √(HRVLF / BPVLF), where HRVLF represents RR-interval variability and BPVLF corresponds to systolic BP variability within the LF range. Coherence of at least 0.5 between systolic BP and R-R intervals within in LF band was observed in all cases. Respiratory frequency was determined manually by calculating the number of breathing cycles per unit time.

### Statistical analysis

We used the student’s t-test for continuous variables and the chi-square test or Fisher’s exact test for nominal variables to compare the baseline characteristics between groups. A mixed-effects model, using the “lmer” package in R, was utilized to analyze baroreflex, as well as HRV and BPV responses between groups and thermal conditions. The analysis estimated within-individual responses over time (from baseline to follow-up). In a subsequent sensitivity analysis, body mass index was added as a covariate to further explore its impact (S2 File). In addition to p-values, standardized effect sizes were calculated to quantify the magnitude of group differences in the response to heat stress. Specifically, linear mixed-effects models were fitted for each outcome with fixed effects of group, time, and their interaction, and a random intercept for participant. Group differences in response to heat stress were assessed using group × time interaction contrasts derived from estimated marginal means. Effect sizes were computed by standardizing these interaction estimates by the residual standard deviation of the corresponding mixed-effects model (i.e., estimate / σ residual), providing a scale-independent measure of effect magnitude appropriate for repeated-measures data (S3 File). Descriptive statistics are presented as means with standard deviations (SD) for continuous variables, and as relative frequencies for categorical variables. For results obtained from the linear mixed-effects models (lmer), estimated marginal means with 95% confidence intervals are reported. Statistical significance was set at p < 0.05. All statistical analyses were conducted using R (release 2022.07.1) [33].

## Results

### Participants characteristics

Participants with T2D were in general heavier and had a greater BMI compared to controls. More than half of people with T2D were using calcium blockers to manage their blood pressure and biguanides for their diabetes care. There were no indications of peripheral artery disease (ABI-measurement) or neuropathy (monofilament test) among the individuals with T2D. The monofilament test showed no significant difference in sensory perception between individuals with T2D and healthy controls. Thermal sensitivity testing showed a decrease in heat sensitivity in the hand in T2D compared with controls, while heat pain detection thresholds did not differ between groups (Table 1).

**Table 1:** Participants characteristics of people with T2D and controls assessed prior to the controlled experiments.

Variable	T2D subjects (n = 10)	Control subjects (n = 10)	P values
**Age, y**	64 ± 4.14	63 ± 5.42	0.649
**Height, cm**	176.2 ± 5.7	176.3 ± 7.1	0.973
**Weight, kg**	93.6 ± 16.3	79 ± 8.19	**0.024**
**BMI, kg/m2**	30.1 ± 4.8	25.4 ± 2.1	**0.015**
**Blood pressure medicationsa**			
Calcium blocker	n = 6		
Beta blocker	n = 1		
Angiotensin II receptor blockers	n = 2		
Angiotensin-converting-enzyme inhibitors	n = 2		
**Diabetes medications**			
Biguanides	n = 7		
SGLT2 Inhibitors	n = 3		
GLP-1 Receptor Agonists	n = 1		
DPP-4 Inhibitors	n = 2		
Insulin & Insulin Analogues	n = 1		
**ABI**	1.14 ± 0.05	1.18 ± 0.05	0.070
**Cold, warm and pain detection thresholds(hand)**			
**CDT**	26.3 ± 4.47	28.8 ± 0.360	**0.025**
**CP**	9.76 ± 6.89	9.92 ± 7.40	0.944
**WDT**	35.7 ± 4.96	32.4 ± 0.700	**0.009**
**HP**	45.9 ± 3.28	45.5 ± 3.12	0.691
**Cold, warm and pain detection thresholds (foot)**			
**CDT**	17.9 ± 10.3	24.7 ± 4.06	**0.012**
**CP**	3.21 ± 4.91	5.59 ± 6.17	0.185
**WDT**	43.3 ± 4.87	41.4 ± 2.41	0.124
**HP**	47.8 ± 1.89	47.7 ± 1.72	0.893
**Monofilament Test**			
**Number of individuals with at least one point with less perception**	6	7	1
**Autonomic Nervous system function**			
**Valsalva Ratio b**	1.35 (0.19)	2,13 (1.18)	0.072
**PRT b**	2.63 (0.77)	1.96 (0.35)	0.064
**30:15 ratio b**	1.16 (0.12)	1.36 (0.73)	0.418
**Paced breathingb**	1.52 (0.32)	1.35 (0.19)	0.125
**Heart rate variability**			
**Average RRI (ms) b**	827.5 (133.43)	955.1 (126.26)	**0.057**
**SD (ms) b**	37.37 (15.06)	34,06 (10.37)	0.606
**RMSSD (ms) b**	34.73 (14.87)	21.74 (11.13)	0.061
**LF (nu) b**	50.44 (20.75)	59.61 (12.31)	0.294
**HF (nu) b**	46.70 (18.04)	40.08 (11.09)	0.382
**LF/HF Ratio b**	1,55 (1,54)	1.70 (0.90)	0.819

Continuous variables are presented as mean values and their SDs.

Abbreviations: BF% body fat percentage; BMI, body mass index; ABI, ankle brachial index test; CDT, Cold Detection Threshold; CP, Cold Pain Threshold; WDT, Warm Detection Threshold; HP, Heat Pain Threshold; PRT, Post-exercise Blood Pressure Recovery Time; 30:15 Ratio, Heart Rate Response to Standing; RRI, RR Interval; SD, Standard Deviation of RR Intervals; RMSSD, Root Mean Square of Successive Differences; LF, Low Frequency (normalized units); HF, High Frequency (normalized units); LF/HF, Low Frequency to High Frequency Ratio.

a Missing information for T2D: Medication; n = 3; b Missing information for T2D; n = 1

There were no differences between the groups in ANS tests or HRV time-domain measures obtained during the baseline assessment day, conducted on a day prior to the heat-controlled experiment day. However, persons with T2D had lower average RR interval and altered frequency-domain parameters, indicating subtle autonomic imbalance (Additional demographic characteristics can be found in the S4 File).

### Exposure to heat

#### Thermal responses.

Skin temperatures were similar between the two groups at baseline but increased less when exposed to heat in subjects with T2D (3.82°C (CI 95%: 3.51,4.13) than in controls (4.51°C (CI 95%:4.22,4.80) (p < 0.05) (The estimated mean for each time point—baseline, heat exposure, and follow-up—along with a comparison of both groups at each time point, are presented in S5 Table of the supplementary material). Heat sensation based on ISO scales showed that the T2D group still felt warm at follow-up after warm exposure, but not in the control group.

#### Heart rate variability, blood pressure variability, and baroreflex sensitivity.

For heart rate and HRV, during heat exposure all responses were smaller in magnitude in T2D. The differences were significant for HR and HF RR, but not for LF RR and LF/HF ratio (Fig 2). Reductions in high-frequency power (HF RR) was 148.15 ms² smaller (p = 0.038, a 54.5% decrease from baseline in T2D vs. a 74% decrease in controls) and low-frequency power (LF RR) was 167.01 ms² smaller (p = 0.063, (a 2.47% decrease from baseline in T2D vs. a 48% decrease in controls) in T2D showing a similar trend. The LF/HF ratio also increased by −2.97 lesser in T2D than in controls, but this difference was not significant (p = 0.186) (a 46% increase from baseline in T2D compared to controls with a 165% increase) (Table 2). The corresponding standardized effect sizes for the group-by-time interaction were −1.83 (95% CI −3.26 to −0.40) for HR, 1.51 (95% CI 0.08 to 2.94) for HF RR, 1.35 (95% CI −0.08 to 2.77) for LF RR, and −0.94 (95% CI −2.37 to 0.48) for LF/HF (S3 File).

**Table 2:** Changes from baseline during whole-body exposure to heat (40°C): heart rate, blood pressure, heart rate variability, blood pressure variability, and baroreflex sensitivity in subjects with T2D and controls, with Group × Time interaction t-values calculated using mixed-effects models.

	Baseline: n = 8 Heat exposure: n = 7	Baseline: n = 10 Heat exposure: n = 9		
	T2D	Control		
	Estimate of change and CI 95% (Exposure -Baseline)	Estimate of change and CI 95% (Exposure -Baseline)	Interaction estimate of group and time and CI 95%	P value for interaction
Heart rate and Heart Rate Variability Frequency-Domain Measures				
HR, (bpm)	5.43 (0.42,10.44)	13.96 (9.53,18.40)	−8.54(−15.22,-1.85)	0.014
HF RR, ms2	−31.21 (−136.0,73.6)	−179.35 (−272.2,-86.5)	148.15 (8.12, 288)	0.038
LF RR, ms2	−1.49 (−134,131.0)	−168.50 (−286,-51.2)	167.01(−9.96,344)	0.063
LF/HF ratio	0.70(−2.67,4.07)	3.67(0.69, 6.66)	−2.97 (−7.47,1.53)	0.186
Blood pressure and Systolic blood pressure variability				
SBP, mmHg	−13.86 (−22.40,-5.33)	−17.86 (−25.413,-10.30)	3.99(−7.41,15.39)	0.479
DPB, mmHg	−1.66(−6.00,2.69)	−5.43 (−9.28,-1.59)	3.78(−2.03,9.58)	0.194
LF BPV, mmHg	2.08(−3.00,7.16)	3.29 (−1.21,7.79)	−1.21 (−7.99,5.58)	0.719
HF BPV, mmHg	0.47 (−1.84,2.77)	2.54 (0.50,4.58)	−2.07(−5.15,1.01)	0.179
Baroreflex sensitivity				
LF BRS, ms/ mmHg	−1.07 (−2.86,0.72)	−3.96 (−5.62,-2.31)	2.89 (0.77,5.20)	0.021
HF BRS, ms/ mmHg	−1.58 (−3.42,0.26)	−4.15 (−5.66,-2.64)	2.58(0.195,4.96)	0.034
Mean BRS, ms/ mmHg	−1.18 (−2.85,0.50)	−4.16 (−5.60,-2.72)	2.99(0.77,5.20)	0.010

Abbreviations: HR, heart rate; LF, low frequency; HF, high frequency; RR, interval; SBP, systolic blood pressure; DBP, diastolic blood pressure; BPV, systolic blood pressure variability; BRS, baroreflex sensitivity

**Fig 2:**
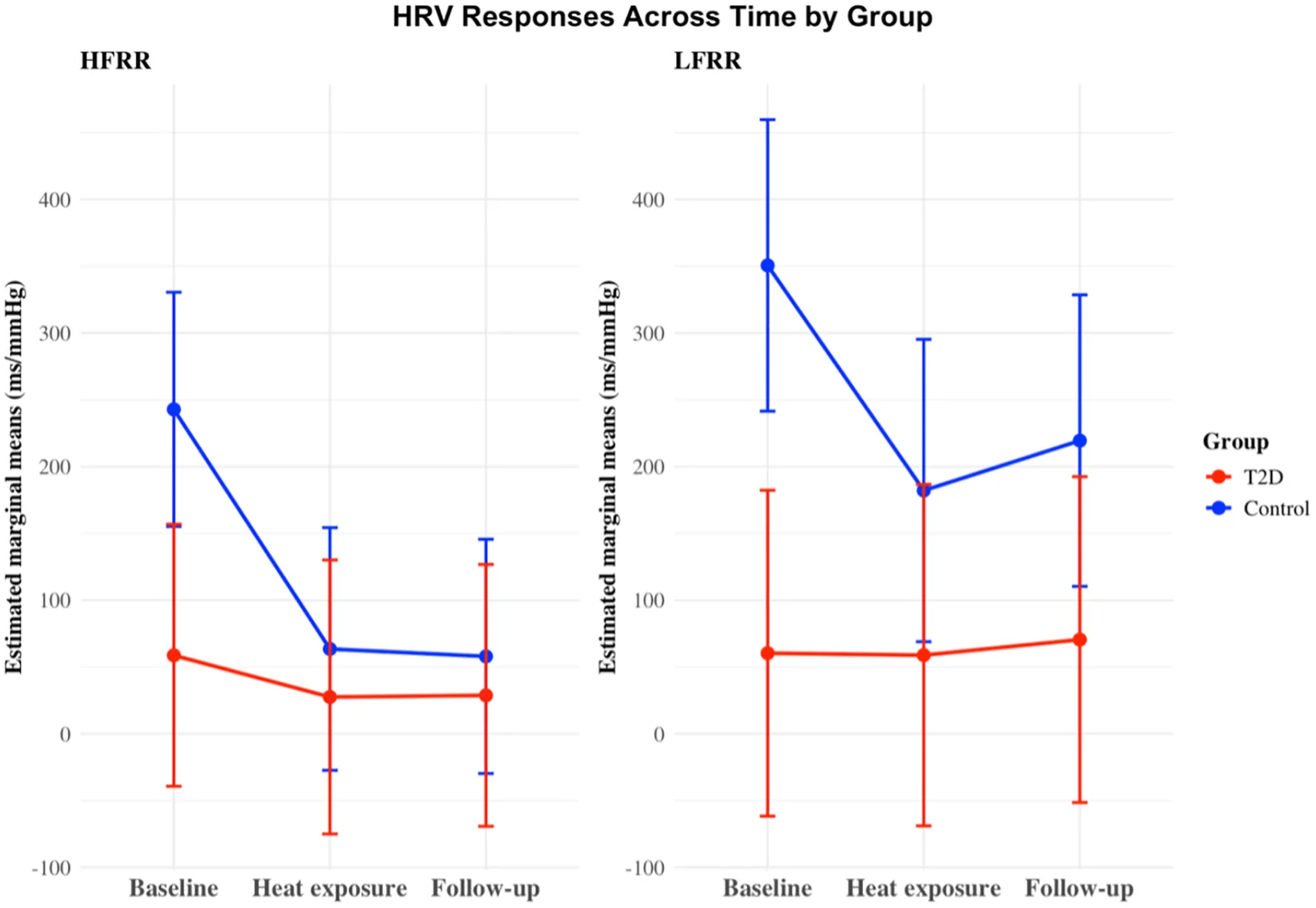
HRV responses across time by group. Data are presented as estimated marginal means with 95% confidence intervals derived from a linear mixed-effects model. Abbreviations: HF, high frequency; LF, low frequency; RR, interval; T2D, Type 2 Diabetes.

For BP and BPV, all parameters showed consistently dampened responses when exposed to heat among T2D compared to controls, though these responses were not significant (Table 2) (Fig 3). Increase in low-frequency BPV (−1.21 mmHg²) and high-frequency BPV (−2.07 mmHg²) were slightly smaller in T2D (p = 0.719 and 0.18, respectively) (Table 2) (LF BPV: + 47.5% in T2D vs. + 49.8% in controls; HF BPV: + 10.6% in T2D vs. + 55.5% in controls). The standardized effect sizes for SBP and DBP were 0.50 (95% CI −0.92 to 1.93) and 0.93 (95% CI −0.50 to 2.36), respectively, while those for LF BPV and HF BPV were −0.25 (95% CI −1.68 to 1.17) and −0.96 (95% CI −2.39 to 0.47), respectively (S3 File).

**Fig 3:**
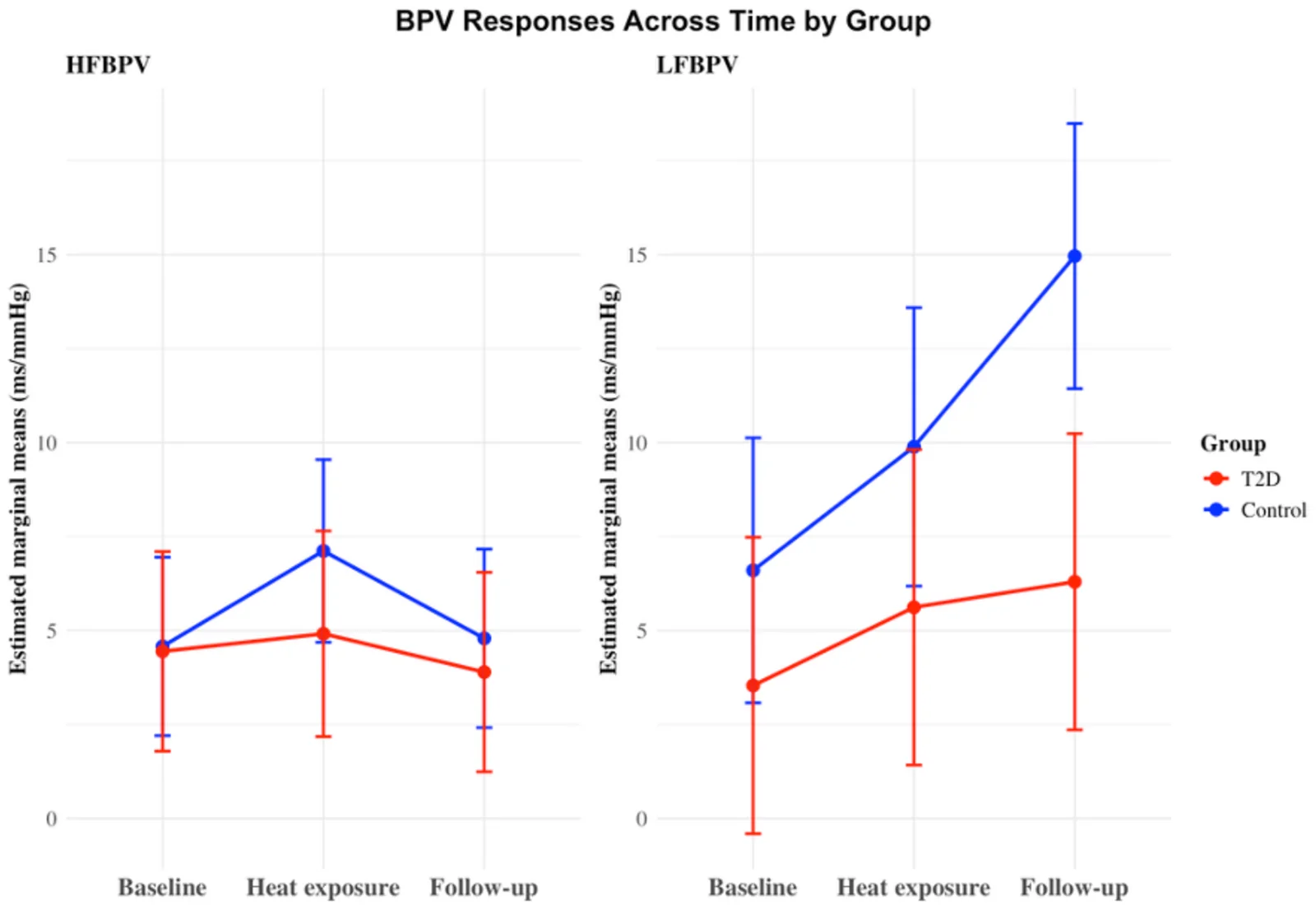
BPV responses across time by group. Data are presented as estimated marginal means with 95% confidence intervals derived from a linear mixed-effects model. Abbreviations: HFBPV, high-frequency blood pressure variability; LFBPV, low-frequency blood pressure variability; T2D, Type 2 Diabetes.

Reductions in LF BRS, HF BRS, and mean BRS were smaller in T2D (2.89, 2.58, and 2.99 ms/mmHg, respectively; p = 0.021, 0.034, and 0.010) compared with controls (Table 2) (Fig 4) (representing a 25%, 35%, and 26% decrease in T2D, compared to a 51%, 62%, and 58% decrease in controls). The standardized effect sizes were 1.73 (95% CI 0.27 to 3.19), 1.62 (95% CI 0.12 to 3.12), and 2.04 (95% CI 0.53 to 3.59) for LF BRS, HF BRS, and mean BRS, respectively (S3 File). The estimated mean for each time point—baseline, heat exposure, and follow-up—along with a comparison of both groups at each time point, can be found in S5 File.

**Fig 4:**
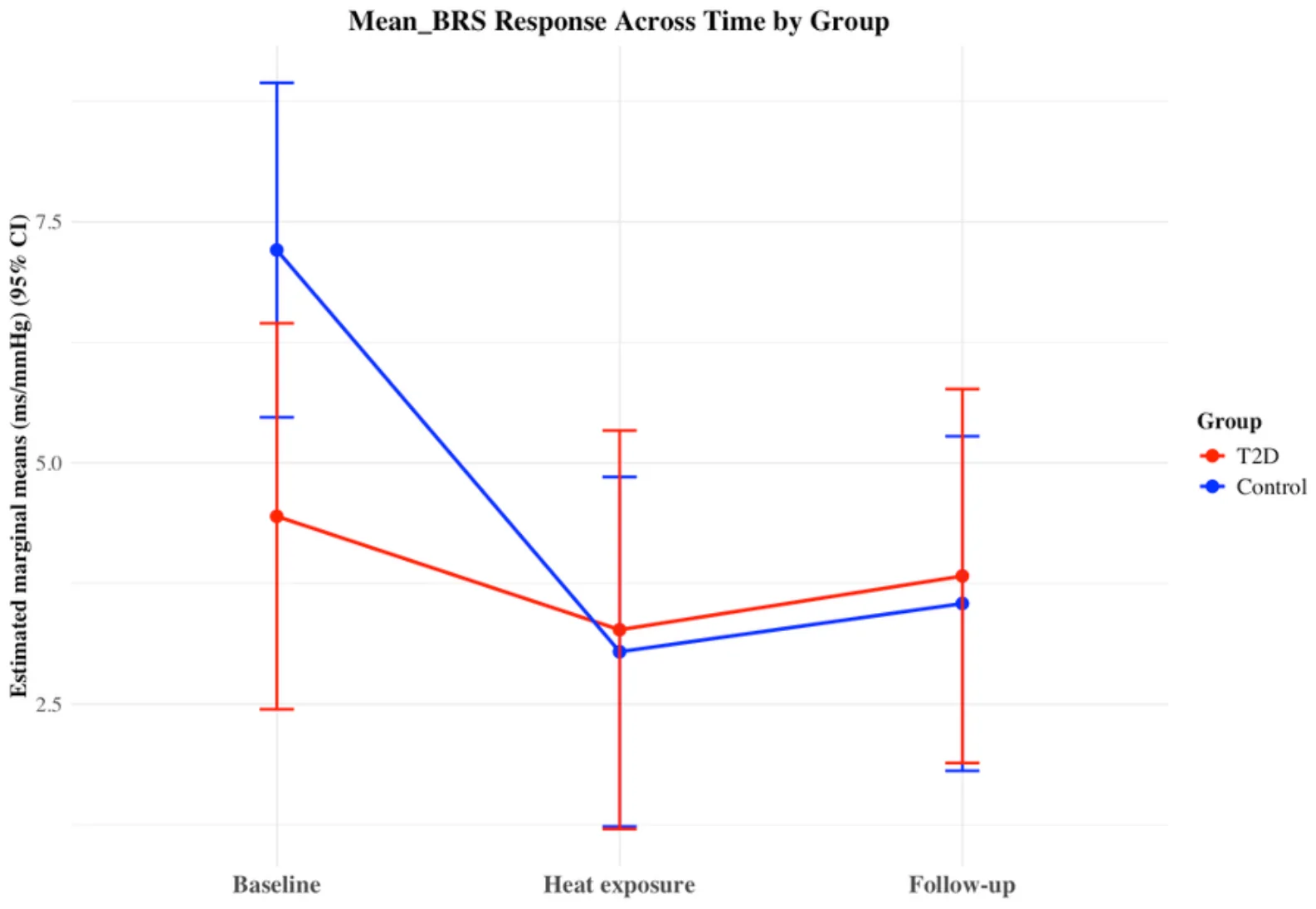
Mean BRS response across time by group. Data are presented as estimated marginal means with 95% confidence intervals derived from a linear mixed-effects model. Abbreviations: BRS, baroreflex sensitivity; T2D, Type 2 Diabetes.

## Discussion

Our study is the first to report baroreflex sensitivity (BRS) and blood pressure variability (BPV), alongside heart rate variability (HRV), in response to whole-body heat exposure in individuals with combined type 2 diabetes and hypertension BRS was reduced less during heat exposure among T2D than in controls. Concerning HVR, all its parameters showed attenuated responses to heat in T2D, but of which only HF RR was statistically significant. Finally, BPV also showed consistent diminished responses during heat, though these were not statistically significant. These findings may indicate that indices of autonomic function are only modestly altered in our T2D participants to heat exposure when compared to healthy participants.

### Characteristics of our population with type 2 diabetes

Based on our thorough clinical assessment, the participants with T2D did not show advanced diseases progression such as peripheral artery disease or neuropathy, though their thermal sensitivity testing revealed decreased warm sensitivity in the hands, consistent with small fibre neuropathy [34]. ANS function, assessed using standard tests before the experiments did not show any major dysfunction among T2D subjects. Only HR and RRSD was higher in T2D, which may indicate sympathetic overactivity [35]. Despite the higher HR observed in individuals with T2D, LF, HF and LH/HF ration did not differ between the groups.

### Heat exposure

Heat exposure caused a significant rise in mean skin temperature (around 4 °C), though this rise was lesser in individuals with T2D. The present study showed that individuals with T2D exhibited a blunted decrease in HRV and BRS, which occurred in parallel with the previously observed blunted cardiovascular responses to this heat exposure [22]. It should also be considered that the T2D group had lower baseline values for HRV and BRS, which may have limited the magnitude of change. In other words, participants with T2D may have been closer to a physiological “floor” effect, thereby constraining a stronger response.

At baseline, participants with T2D exhibited lower HRV parameters (LF RR and HF RR) compared with controls, consistent with the overall reduction in HRV commonly reported in diabetes [36]. LF power reflects both sympathetic and parasympathetic activity, including baroreflex-mediated blood pressure regulation [37], while HF primarily represents parasympathetic activity associated with respiration [38]. The LF/HF ratio is also often used as a rough indicator of autonomic balance, with higher values suggesting sympathetic dominance, though its accuracy is debated due to overlapping influences on LF and HF components [37,39]. During heat exposure, both groups exhibited reductions in HF RR, reflecting parasympathetic withdrawal consistent with the expected physiological response to the heat [40–42]. However, this response was smaller in the T2D group. This blunted autonomic adjustment in T2D could suggest reduced flexibility in modulating sympathovagal balance under thermal stress [40,41]. Carrillo et al. reported similar patterns in a study examining the effects of prolonged heat exposure (3-h duration) on HRV and indices of autonomic function in young healthy adults, as well as older adults with or without T2D with similar mean age (63 ± 5 years old) to our population. Their findings revealed that older individuals with or without T2D exhibited lower baseline HRV and a blunted autonomic response to heat stress compared to the younger group, reinforcing that age—more than diabetic status—attenuates autonomic responses to environmental extremes [20]. Support for impaired autonomic nervous system function in middle-aged to older adults (50–70 years) with T2D was reported by Macartney et al. (2021). [43]. In their study, a 7-day exercise–heat acclimation protocol reduced exercising heart rate in participants with T2D; however, HRV did not improve. The absence of change in HRV despite a lower heart rate at exercise suggests persistent autonomic dysfunction in this age group, which may impair cardiovascular regulation and potentially compromise thermoregulatory capacity [43].

Our results show that both LF BRS and mean BRS were significantly lower at baseline in T2D compared to controls. In addition, these measures decreased less in response to heat stress in T2D. This pattern indicates reduced baroreflex sensitivity, which impairs cardiovascular autonomic and blood pressure regulation [44]. Since the baroreflex regulates short-term blood pressure stability by adjusting heart rate and vascular tone, this attenuated response suggests a diminished ability of individuals with T2D to maintain cardiovascular stability under heat exposure [22,45–47]. Concerning BPV, our results also showed a lesser rise in both LF BPV and HF BPV from baseline to heat exposure in T2D (not significant). This response may reflect diminished autonomic co-activation to regulate BP and HR, perhaps due to reduced baroreflex sensitivity observed in T2D.

Notably, standardized effect sizes were large for HR, HRV indices, and all BRS measures. Among BP and BPV measures, effect sizes ranged from small (LF BPV) to large (DBP and HF BPV), despite several of these outcomes not reaching conventional levels of statistical significance. Although confidence intervals were wide due to the small sample size, these estimates suggest that the observed differences in autonomic regulation between groups during heat exposure may be physiologically meaningful.

In summary, individuals with type 2 diabetes exhibited a blunted autonomic response to acute heat exposure, implying reduced physiological flexibility during thermal stress in this population. However, the relatively modest differences between groups could be due to the fact that those with T2D were not severely affected by the disease, consistent with the benign neurophysiological assessments.

### Possible mechanisms explaining the observed responses to thermal stress in T2D

The observed attenuated responses related to autonomic nervous system and cardiovascular regulation in participants with T2D may be influenced by multiple mechanisms associated with the disease progress and related pathology. Impaired baroreflex sensitivity blunts rapid adjustments of heart rate and blood pressure, limiting the heart’s ability to respond to thermal stress [45,47]. Arterial stiffness and vascular remodelling related to combined T2D and hypertension can further limit the ability to modulate blood pressure effectively, while microvascular rarefaction reduces peripheral blood flow and limits tissue perfusion during temperature challenges [48]. In addition, endothelial dysfunction, common in T2D, reduces nitric oxide availability, impairing cutaneous vasodilation and vasoconstriction, thus compromising blood flow redistribution [49]. Also, the degree of autonomic neuropathy and particularly small-fibre involvement could influence cardiovascular and thermoregulation [50]. Finally, it should be noted that several other bodily systems (metabolic, renal, inflammatory) can be affected by T2D and influence the observed neural and cardiovascular responses to thermal stress, but these were not assessed in the present study.

### Strengths and limitations

One of the key strengths of this study is the recruitment of participants from the general population, rather than from a specialized clinical group, which enhances the generalizability of the findings. Another major strength of this study is that all participants underwent identical, carefully controlled conditions and measurement procedures, with autonomic and cardiovascular responses to whole-body heat. To minimize the influence of confounding variables related to vascular health, such as smoking, only non-smokers were included in the study. In addition, we conducted a sensitivity analysis which did not support an influence of BMI on the obtained results. However, there are also some limitations to consider. To ensure participant safety, all individuals continued their usual medications, including those prescribed for hypertension and diabetes. As a result, our findings reflect cardiovascular responses in individuals undergoing treatment, rather than untreated cases. We acknowledge that these medications can affect autonomic regulation, including heart rate variability and baroreflex sensitivity. Beta-blocker therapy may significantly affect heart rate variability, including frequency-domain measures such as the HF component. The small sample size, coupled with the use of a beta-blocker drug by one participant, may have influenced HRV and baroreflex estimates and potentially biased group-level comparisons. The widespread use of multiple medications among participants also makes it difficult to isolate the effects of any one drug. Moreover, the frequent coexistence of hypertension with T2D further complicates the ability to distinguish the independent mechanisms of each condition. Therefore, we recommend that future studies include a hypertensive non-diabetic comparison group, as well as a group with T2D without hypertension, to clarify the independent contributions of these conditions. Nevertheless, this methodology mirrors the typical clinical profile of individuals with T2D and hypertension who are under treatment. We also did not measure core temperature, which could have provided additional insights in to the degree of heat strain and associated autonomic responses. Moreover, we did not assess renal function, inflammatory markers, or comprehensive hematological profiles that may influence autonomic and cardiovascular responses in T2D. Lastly, the inclusion of female participants could have further strengthened the interpretation of our findings.

## Conclusions

The present study indicates that individuals with T2D generally exhibit diminished autonomic nervous function and cardiovascular regulation responses during environmental heat exposure. The observed findings suggest impaired modulation of cardiovascular responses to heat exposure that could increase thermal strain of persons with T2D. These findings highlight the importance of a comprehensive approach in future research, integrating autonomic, vascular, inflammatory, and metabolic assessments to better understand how T2D affects the body’s adaptation to environmental stressors.

## Supporting information

S1 File Autonomic nervous system methods and protocols including Valsalva maneuver, tilt test, paced breathing, and HRV analysis. (DOCX)

S2 File Heart rate, blood pressure, heart rate variability, blood pressure variability, and baroreflex sensitivity in subjects with T2D and controls in response to whole-body heat exposure (+40 °C) at rest, adjusted for Body Mass Index. (DOCX)

S3 File Estimates and standardized effect sizes for group × time interaction during heat exposure. (DOCX)

S4 File Participants characteristics of people with T2D and controls prior to the controlled experiment. (DOCX)

S5 File Heart rate, blood pressure, heart rate variability, blood pressure variability, baroreflex sensitivity, and skin temperature during heat exposure. (DOCX)
