# Supplementary material for: Heart rate and blood pressure variability, and baroreflex sensitivity, during heat exposure in persons with type 2 diabetes and hypertension
Source: PLoS One. 2026 Oct 27;21(10):e0361367. doi: 10.1371/journal.pone.0361367 (PMC13712578; doi:10.1371/journal.pone.0361367)
Supplement: Supplementary file 1 [file pone.0361367.s001.docx]

**Supplement 1**

**More detailed procedures and calculations related to the autonomic nervous system (ANS) parameters**

#### The autonomic nervous system (ANS) parameters

Baseline ANS measurements were conducted at the neurophysiological clinic at the university hospital of Oulu prior to the experiment day.

#### The Valsalva maneuver

The Valsalva maneuver was performed with the patient seated, using a bugle equipped with an air leak to ensure an open glottis. Patients were instructed to take a deep breath and maintain an expiratory pressure of 40 mm Hg for 15 seconds, measured using a pressure gauge attached to a 5- to 10-mL plastic syringe. They were first given the opportunity to practice the maneuver until they were comfortable with the procedure, followed by rest to allow recovery of the cardiovascular functions before the first recorded trial. The maneuver was repeated until two consistent blood pressure responses were obtained. Heart rate and blood pressure were continuously monitored using an electrocardiographic monitor and a noninvasive beat-to-beat blood pressure device, respectively.

The Valsalva maneuver consists of four phases that reflect dynamic changes in heart rate and blood pressure in response to altered intrathoracic pressure. Phase I involves a brief rise in blood pressure at the onset of the respiratory strain. Phase II is characterized by a fall in blood pressure due to decreased venous return, followed by a compensatory sympathetic-mediated vasoconstriction. Phase III occurs with the release of intrathoracic pressure, leading to a transient blood pressure drop. Phase IV features a rebound overshoot in blood pressure due to restored cardiac output and persistent vasoconstriction (1). Blood pressure recovery time (PRT) was defined as the interval from the nadir of systolic blood pressure at the end of Phase III to the return to baseline levels. It reflects the time required for sympathetic vasoconstriction to normalize blood pressure after transient hypotension. As such, PRT is a useful marker of adrenergic function and can reveal progressive autonomic dysfunction, even in cases where Phase II responses are blunted or absent (1, 2). In addition, the Valsalva ratio (VR) was calculated by dividing the maximum heart rate during the blood pressure drop by the minimum heart rate following the subsequent rebound (3).

#### 30:15 ratio

The patient was passively tilted from supine to an upright position of 80° over 5 to 10 seconds using a motorized table. The “30:15 ratio” was calculated by dividing the longest RR interval, occurring around beat 30 (within beats 20–40), by the shortest RR interval, occurring around beat 15 (within beats 10–20), following a rapid passive upright tilt (2 seconds to 80°). Systolic and diastolic blood pressure responses to the tilt were recorded continuously, as well as at 0, 2, and 5 minutes after tilting. The greatest decrease (or smallest increase) in systolic and diastolic blood pressure throughout the tilting procedure was also identified (3). Physiologically, a 30:15 ratio >1.04 is considered normal (4).

#### Paced breathing

Heart rate was monitored from an electrocardiogram using electrodes placed in standard positions, and R–R intervals were converted to heart rate. Respiratory activity was recorded using chest bellows connected to a pressure transducer or a nasal thermistor. The patient was positioned supine and instructed to relax while a 1-minute baseline was recorded. This was immediately followed by six cycles of metronomic breathing, with 5 seconds each for inhalation and exhalation. Patients were instructed to breathe as deeply and slowly as possible, avoiding sudden breaths, breath-holding, or rapid inhalations and exhalations. After a 2-minute rest, the procedure was repeated.

During paced breathing, R–R intervals were analyzed by calculating the ratio of the longest (during expiration) to the shortest (during inspiration) R–R interval over five consecutive breathing cycles (3). The median of these ratios was determined. The test was performed twice, and the higher median R–R interval ratio from the two trials was recorded as the “maximum–minimum ratio.” Endotidal CO₂ was monitored when necessary to rule out hyperventilation. To help patients maintain consistent breathing rhythm, visual cues (such as an oscillating bar or sine wave) or auditory cues provided by the technician using a stopwatch were used (5).

#### Heart rate variability

Heart rate variability (HRV) was analyzed using electrocardiographic recordings obtained during a 5-minute resting period in the supine position, following a 30-minute rest period preceding the tests. The average R–R interval (RRI) in milliseconds represented the mean duration between consecutive heartbeats. The standard deviation (SD) of R–R intervals provided a measure of overall variability in heart rate. The root mean square of successive differences (RMSSD) between adjacent R–R intervals was calculated as an indicator of short-term, high-frequency variations primarily influenced by parasympathetic activity. From these recordings, both time-domain and frequency-domain parameters were calculated. In the frequency domain, low-frequency (LF) and high-frequency (HF) components were expressed in normalized units (nu) to allow for meaningful comparison between sympathetic and parasympathetic contributions. The LF component, typically associated with both sympathetic and parasympathetic modulation, and the HF component, predominantly reflecting parasympathetic tone, were used to derive the LF/HF ratio, which served as an index of sympathovagal balance (6, 7).

# References

1.	Goldstein DS, Cheshire WP, Jr. Beat-to-beat blood pressure and heart rate responses to the Valsalva maneuver. Clin Auton Res. 2017;27(6):361-7.

2.	Vogel ER, Sandroni P, Low PA. Blood pressure recovery from Valsalva maneuver in patients with autonomic failure. Neurology. 2005;65(10):1533-7.

3.	Cheshire WP, Freeman R, Gibbons CH, Cortelli P, Wenning GK, Hilz MJ, et al. Electrodiagnostic assessment of the autonomic nervous system: A consensus statement endorsed by the American Autonomic Society, American Academy of Neurology, and the International Federation of Clinical Neurophysiology. Clin Neurophysiol. 2021;132(2):666-82.

4.	Ziemssen T, Siepmann T. The Investigation of the Cardiovascular and Sudomotor Autonomic Nervous System-A Review. Front Neurol. 2019;10:53.

5.	Chow KE, Dhyani R, Chelimsky TC. Basic Tests of Autonomic Function. J Clin Neurophysiol. 2021;38(4):252-61.

6.	Shaffer F, Ginsberg JP. An Overview of Heart Rate Variability Metrics and Norms. Front Public Health. 2017;5:258.

7.	Zygmunt A, Stanczyk J. Methods of evaluation of autonomic nervous system function. Archives of Medical Science. 2010;6(1):11-8.
