# Supplementary material for: Heart rate and blood pressure variability, and baroreflex sensitivity, during heat exposure in persons with type 2 diabetes and hypertension
Source: PLoS One. 2026 Oct 27;21(10):e0361367. doi: 10.1371/journal.pone.0361367 (PMC13712578; doi:10.1371/journal.pone.0361367)
Supplement: Supplementary file 2 [file pone.0361367.s002.docx]

**Supplement 2**

Heart Rate, Blood Pressure, Heart Rate Variability, Blood Pressure Variability, and Baroreflex Sensitivity in Subjects with T2D and Controls in Response to Whole-Body Heat Exposure (+40 °C) at Rest, Adjusted for Body Mass Index. The values represent Estimated Marginal Means (95% Confidence Interval) Calculated by Mixed-Effect Models.

|  | **T2D** | | | **Control** | | | **P-value for**  **T2D vs control** | | |
| --- | --- | --- | --- | --- | --- | --- | --- | --- | --- |
| **Variable** | **Baseline (n=8)** | **Exposure (n=7)** | **Follow-up  (n=8)** | **Baseline (n=10)** | **Exposure (n=9)** | **Follow-up  (n=10)** | **Baseline** | **Exposure** | **Follow-up** |
| Hear rate and Heart Rate Variability Frequency-Domain Measures | | | | | | | | | |
| HR | 77(69.2,84.8) | 82.5(74.6,90.3) | 77.8(70,85.5) | 65.3(58.6,72.1)^ab^ | 79.3(72.5,86.2) | 74.7(68,81.4) | 0.03 | 0.56 | 0.56 |
| LF RR, ms^2^ | 73.4(-60.2,207) | 70.8(-66.8 ,208) | 83.6(-50,217) | 341 (225,458)^a^ | 172.3(51.6,293) | 210.2(93.9,327) | 0.00 | 0.29 | 0.17 |
| HF RR, ms^2^ | 69.8(-37.7 ,177) | 37.7(-72.9,148) | 39.8(-67.7, 147) | 235.1(141.5,329) ^ab^ | 55.2(-41.7,152) | 50.2(-43.4,144) | 0.03 | 0.82 | 0.88 |
| LF/HF | 1.93(-1.19,5.05) | 2.59(-0.64,5.82) | 3.98(0.86,7.10) | 1.94(-0.78,4.66) ^ab^ | 5.59(2.73,8.43) | 6.75(4.02,9.47) | 0.99 | 0.18 | 0.20 |
| Blood pressure and Systolic blood pressure variability | | | | | | | | | |
| SBP ,mmHg | 120(109.1,131)^a^ | 106(95.2,117) | 115(104,126) | 130(120.7,140)^ac^ | 112(102.7,122) | 122(113.1,132) | 0.17 | 0.42 | 0.31 |
| DBP ,mmHg | 73.1(68.5,77.6) | 64(58.7,69.4) | 66.1(60.9,71.3) | 75.4(70.9,79.9)^a^ | 70 (65.3, 74.6) | 73.1(68.5,77.6) | 0.011 | 0.11 | 0.06 |
| LF BPV, mmHg | 3.79(-0.45,8.04) | 5.85(1.42,10.29) | 6.55(2.30,10.80) | 6.42(2.70,10.14) ^b^ | 9.68(5.77,13.60) | 14.78(11.06,18.50) | 0.37 | 0.21 | 0.00 |
| HF BPV, mmHg | 4.58(1.61,7.56) | 5.05(2.03,8.08) | 4.03(1.06,7.01) | 4.48(1.90,7.06) ^a^ | 7.02(4.39,9.66) | 4.69(2.12,7.27) | 0.96 | 0.34 | 0.74 |
| Baroreflex sensitivity | | | | | | | | | |
| LF BRS, ms/ mmHg | 4.77 (2.96,6.58) | 3.67(1.81,5.54) | 4.55(2.74,6.37) | 7.40(5.83,8.97) ^ab^ | 3.40(1.70,5.10) | 3.65(2.08, 5.22) | 0.04 | 0.83 | 0.46 |
| HF BRS, ms/ mmHg | 5.16(2.61,7.70) | 3.59(0.98,6.19) | 4.30(1.80,6.81) | 6.24(4.11,8.37)^a b^ | 2.08(-0.09,4.25) | 2.66(0.53,4.79) | 0.57 | 0.39 | 0.34 |
| Mean BRS, ms/ mmHg | 5.05(2.92,7.17) | 3.87(1.69,6.06) | 4.46 (2.38,6.55) | 6.83(5.06,8.60)^ab^ | 2.64(0.78,4.49) | 3.17(1.4,4,94) | 0.21 | 0.41 | 0.36 |

Values are group means and 95% confidence interval. HR, heart rate; LF, low frequency; HF, high frequency; RR, interval; SBP, systolic blood pressure; DBP, diastolic blood pressure; BPV, systolic blood pressure variability; BRS, baroreflex sensitivity, °C; degrees Celsius.

^a^ P<0.05 baseline vs. exposure within that group; ^b^ P<0.05 baseline vs. follow-up within that group; ^c^ P<0.05 exposure vs. follow-up within that group.
