# Supplementary material for: Heart rate and blood pressure variability, and baroreflex sensitivity, during heat exposure in persons with type 2 diabetes and hypertension
Source: PLoS One. 2026 Oct 27;21(10):e0361367. doi: 10.1371/journal.pone.0361367 (PMC13712578; doi:10.1371/journal.pone.0361367)
Supplement: Supplementary file 3 [file pone.0361367.s003.docx]

**Supplement 3.**

Estimates and standardized effect sizes ($d$) for the interaction of group and time (Heat Exposure vs. Baseline) for Heart Rate, Blood Pressure, Heart Rate Variability, Blood Pressure Variability, Baroreflex Sensitivity to whole-body exposure to heat (+40 °C) at rest: in T2D and control groups.

| **Outcome** | **Estimate (95% CI)** | **d(95% CI)** |
| --- | --- | --- |
| HR | -8.54(-15.22,-1.85) | -1.83 ( -3.26 -0.40) |
| HF_FFT | 148.15 (8.12, 288) | 1.51 (0.08 , 2.94) |
| LF_FFT | 167.01(-9.96,344) | 1.35( -0.08 , 2.77) |
| LF/HF | -2.97 (-7.47,1.53) | -0.94 (-2.37 , 0.48) |
| SBP | 3.99(-7.41,15.39) | 0.5( -0.92 ,1.93) |
| DBP | 3.78(-2.03,9.58) | 0.93( -0.50, 2.36) |
| LF_BPV | -1.21 (-7.99,5.58) | -0.25[(-1.68, 1.17) |
| HF_BPV | -2.07(-5.15,1.01) | -0.96( -2.39, 0.47) |
| LF_BRS | 2.89 (0.77,5.20) | 1.73(0.27, 3.19) |
| HF_BRS | 2.58(0.195,4.96) | 1.62(0.12, 3.12) |
| MEAN_BRS | 2.99(0.77,5.20) | 2.04(0.53 ,3.59) |

HR, heart rate; LF, low frequency; HF, high frequency; RR, interval; SBP, systolic blood pressure; DBP, diastolic blood pressure; BPV, systolic blood pressure variability; BRS, baroreflex sensitivity.
