# Supplementary material for: Heart rate and blood pressure variability, and baroreflex sensitivity, during heat exposure in persons with type 2 diabetes and hypertension
Source: PLoS One. 2026 Oct 27;21(10):e0361367. doi: 10.1371/journal.pone.0361367 (PMC13712578; doi:10.1371/journal.pone.0361367)
Supplement: Supplementary file 4 [file pone.0361367.s004.docx]

**Supplement 4.**

Participants characteristics of people with T2D and controls prior to the controlled experiment.

| **Variable** | **T2D subjects (n= 10)** | **Control subjects (n=10)** | **P values** |
| --- | --- | --- | --- |
| **Fat percentage (%)** | 31.6 ± 6.5 | 25.2 ± 3.7 | 0.017 |
| **Health perception** |  |  | 0.04 |
| Very good | 12.5% (n=1) | 30 % (n=3) |  |
| Fairly good | 25 % (n=2) | 60 % (n=6) |  |
| Average | 50 % (n=4) | 10 % (n=1) |  |
| Somewhat poor | 12.5 % (n=1) | - |  |
| **Alcohol consumption %** |  |  | 0.58 |
| At least once a month. | 75 % (n=6) | 60% (n=6) |  |
| Less than once a month | 25% (n=2) | 10% (n=1) |  |
| Stopped drinking | - | 20% (n=2) |  |
| Never used alcohol | - | 10% (n=1) |  |
| **Physical activity during leisure time** |  |  | 0.17 |
| Physical activity like running, at least four hours per week. | 25% (n=2) | 50% (n=5) |  |
| Physical activity like running, average three hours per week. | 37.5% (n=3) | 50% (n=5) |  |
| No physical activity | 37.5% (n=3) | - |  |
| **Physical activity at work** |  |  | 0.25 |
| Working involves mainly sitting | 85.7% (n=6) | 55.6% (n=5) |  |
| Walking or caring heavy items | - | 33.3% (n=3) |  |
| Walking, lifting, and caring heavy items and climbing stairs | 14.3% (n=1) | 11.1% (n=1) |  |

^a^ Missing information for T2D: Health perception; n=2, Alcohol consumption %, n=2; Physical activity during leisure time, n=2; Physical activity during work time, n=3

^b^ Missing information for control: Physical activity during work time, n=1
