# Supplementary material for: Heart rate and blood pressure variability, and baroreflex sensitivity, during heat exposure in persons with type 2 diabetes and hypertension
Source: PLoS One. 2026 Oct 27;21(10):e0361367. doi: 10.1371/journal.pone.0361367 (PMC13712578; doi:10.1371/journal.pone.0361367)
Supplement: Supplementary file 5 [file pone.0361367.s005.docx]

**Supplement 5.**

Heart Rate, Blood Pressure, Heart Rate Variability, Blood Pressure Variability, Baroreflex Sensitivity and skin temperature in subjects with T2D and controls in response to whole-body exposure to heat (+40 °C) at rest: The values represent Estimated Marginal Means (95% Confidence Interval) Calculated by Mixed-Effect Models.

|  | **T2D** | | | **Control** | | | **P-value for**  **T2D vs control** | | |
| --- | --- | --- | --- | --- | --- | --- | --- | --- | --- |
| **Variable** | **Baseline (n=8)** | **Exposure (n=7)** | **Follow-up  (n=8)** | **Baseline (n=10)** | **Exposure (n=9)** | **Follow-up  (n=10)** | **Baseline** | **Exposure** | **Follow-up** |
| Heart rate and Heart Rate Variability Frequency-Domain Measures | | | | | | | | | |
| HR | 79.7(72.2, 87.1) | 85.1(77.5  , 92.7) | 80.4(73.0, 87.9) | 63.5(56.8,70.2)^ab^ | 77.4(70.7, 84.2) | 72.8(66.1, 79.5) | 0.00 | 0.13 | 0.13 |
| LF RR, ms^2^ | 60.3(-61.6,182) | 58.9(-68.9 ,187) | 70.5(-51.5,192) | 350.6(241.5,46)^a^ | 182,1(68.9, 295) | 219,4(110.3,329) | 0.00 | 0.15 | 0.07 |
| HF RR, ms^2^ | 42.82(-39.3 ,157) | 19.49(-75.0,130) | 21.65(-69.3, 127) | 242.8(155.2,33) ^ab^ | 63.5(-27.3, 154) | 57.9(-29.7,146) | 0.00 | 0.58 | 0.65 |
| LF/HF | 1.53(-1.36,4.41) | 2.23(-0.817,5.27) | 3.58(0.692,6.46) | 2.23(-0.36,4.81) ^ab^ | 5.90(3.21, 8.59) | 7.03(4.45,9.61) | 0.38 | 0.79 | 0.62 |
| Blood pressure and Systolic blood pressure variability | | | | | | | | | |
| SBP ,mmHg | 122(112.4,133)^a^ | 109(98.3 ,119) | 117(107.3,127) | 128(119.3,137)^ac^ | 110(101.3,120) | 121(111.7 ,130) | 0.38 | 0.79 | 0.62 |
| DBP ,mmHg | 67.3(62.30,72.3) | 65.7(60.5,70.8) | 67.7(62.7, 72.7) | 74.2(69.7,78.7)^a^ | 68.8(64.2, 73.3) | 71.9(67.4 ,76.4) | 0.04 | 0.36 | 0.21 |
| LF BPV, mmHg | 3.22(-0.40,7.48) | 4.75(1.42, 9.81) | 4.87(2.36,10.24) | 6.60(3.07,10.13) ^b^ | 9.89(6.18,13.59) | 14.96(11.44,18.49) | 0.25 | 0.13 | 0.00 |
| HF BPV, mmHg | 4.45(1.79, 7.10) | 4.92(2.18 ,7.65) | 3.90(1.24,6.55) | 4.58(2.20,6.95) ^a^ | 7.12(4.69,9.55) | 4.79(2.42,7.17) | 0.94 | 0.23 | 0.61 |
| Baroreflex sensitivity | | | | | | | | | |
| LF BRS, ms/ mmHg | 4.25 (2.5 ,5.98) | 3.19(1.39, 4.99) | 4.04(2.31,5.76) | 7.75(6.21,9.30) ^ab^ | 3.79(2.13, 5.45) | 4.01 (2.4, 5.55) | 0.00 | 0.62 | 0.98 |
| HF BRS, ms/ mmHg | 4.51(2.142 ,6.88) | 2.93(0.50,5.37) | 3.62(1.317,5.93) | 6.66(4.59,8.72)^a b^ | 2.51(0.41, 4.60) | 3.08(1.02,5.14) | 0.17 | 0.79 | 0.72 |
| Mean BRS, ms/ mmHg | 4.45(2.45,6.45) | 3.27(1.21, 5.34) | 3.83 (1.89,5.77) | 7.21 (5.47,8.94)^ab^ | 3.04(1.23, 4.85) | 3.54(1.81,5.28) | 0.04 | 0.87 | 0.82 |
| Skin temperature | | | | | | | | | |
| Mean skin temperature (°C) | 31.9 (31.6, 32.2) | 35.7(35.4,36.1) | 33.80(33.5 ,34.1) | 31.8 0 (31.5, 32.1) | 36.3 (36.0 ,36.6) | 34.1 (33.8,34.4) | 0.52 | 0.02 | 0.19 |

Values are group means and 95% confidence interval. HR, heart rate; LF, low frequency; HF, high frequency; RR, interval; SBP, systolic blood pressure; DBP, diastolic blood pressure; BPV, systolic blood pressure variability; BRS, baroreflex sensitivity, °C; degrees Celsius.

^a^ P<0.05 baseline vs. exposure within that group; ^b^ P<0.05 baseline vs. follow-up within that group; ^c^ P<0.05 exposure vs. follow-up within that group.
